# Progress in MXenes and Their Composites as Electrode Materials for Electrochemical Sensing and Dye-Sensitized Solar Cells

**DOI:** 10.3390/molecules29225233

**Published:** 2024-11-05

**Authors:** Sanjeevamuthu Suganthi, Khursheed Ahmad, Tae Hwan Oh

**Affiliations:** School of Chemical Engineering, Yeungnam University, 280 Daehak-Ro, Gyeongsan 38541, Republic of Korea

**Keywords:** MXenes, composites, phenol sensors, electrode materials, dye-sensitized solar cells

## Abstract

In the present mini-review article, we have compiled the previously reported literature on the fabrication of MXenes and their hybrid composite materials based electrochemical sensors for the determination of phenolic compounds and counter electrodes for platinum (Pt)-free dye-sensitized solar cells (DSSCs). MXenes are two-dimensional (2D) materials with excellent optoelectronic and physicochemical properties. MXenes and their composite materials have been extensively used in the construction of electrochemical sensors and solar cell applications. In this paper, we have reviewed and compiled the progress in the construction of phenolic sensors based on MXenes and their composite materials. In addition, co1.unter electrodes based on MXenes and their composites have been reviewed for the development of Pt-free DSSCs. We believe that the present review article will be beneficial for the researchers working towards the development of phenolic sensors and DSSCs using MXenes and their composites as electrode materials.

## 1. Introduction

In the past few years, a new class of layered materials has been classified as MXenes; they possess excellent unique chemical, mechanical, superior conductivity, and optoelectronic properties [1,2,3,4]. MXenes are two-dimensional (2D) materials and consist of metal carbides or metal nitrides with a general molecular formula of M_n+1_X_n_T_x_ [5,6,7]. In 2011, Naguib reported the synthesis of titanium carbide (Ti_3_C_2_) MXene using novel strategies [8]. MXenes possess excellent properties, which motivated the researchers to explore their potential in various applications such as sensors, energy storage devices, solar cells, batteries, and oxygen or hydrogen evolution reactions [9,10,11]. MXenes and graphene derivatives possess similar properties, but MXenes have metallic conductivity that is superior to that of graphene or graphene derivatives [12,13]. This may significantly facilitate electron transfer and improve electrochemical activity for redox reactions [14]. In addition, MXenes have terminal functional groups such as –OH, –O, and –F, which can be tailored during synthetic processes, and such functional groups may improve the hydrophilic nature of MXenes and provide more active sites for electrochemical reactions [15]. The layered structure of MXenes may allow for the intercalation of ions and improve electro-catalytic properties for electrochemical applications [16]. This property is less pronounced in graphene or graphene derivatives, where intercalation can be more challenging due to their planar structure [12,17]. In addition, MXenes can also be easily exfoliated into single or a few layered structures, enabling tunable properties based on the number of layers [18,19].

Environmental pollution and the energy crisis are the two major concerns of our contemporary society, and they need significant attention [20,21]. The ongoing expansion of industrialization and rapid technological progress have profoundly affected the environment across different regions globally [22,23,24]. The increasing pollution levels in the air, soil, and water present serious threats to the health and well-being of populations around the world, leading to widespread repercussions [25,26,27]. 

Numerous toxic materials and hazardous compounds—particularly phenolic compounds such as phenol [28], chlorophenol [29], bisphenol A [30], resorcinol [31], nitrophenol [32], catechol [33], hydroquinone [34], etc.—are widely used in a variety of applications. Phenolic compounds have a vital role in the development of the industrial sector [35]. These compounds are used in the manufacturing and production of plastics, textiles, petrochemical products, resins, and dyes [36,37,38]. These compounds may be released into the environment in the form of byproducts and contaminants [37]. Long-term/excessive exposure to high levels of phenolic compounds may cause damage to the heart, kidneys, and liver [39]. Thus, it is necessary to monitor the presence of phenolic compounds to avoid negative impacts on human beings and aquatic life [40]. Previously, various methods such as liquid chromatography (LC), gas chromatography (GC), and capillary electrophoresis (CE) were extensively used for the determination of phenolic compounds [41,42,43,44,45]. Despite the decent performance of these conventional methods, there are some major drawbacks, such as high cost, complex and large areas for implantation, the need for highly expert operators, and the long time needed for sample determination/preparation, which have prompted researchers to develop other alternative methods. In this regard, electrochemical methods have received enormous attention because of their simplicity, low cost, high sensitivity, portability, selectivity, and stability [46,47,48]. It is believed that the performance of electrochemical sensors is largely dependent on the physicochemical properties of electro-catalysts (also known as electrode modifiers/materials). The selection of efficient electrode materials is crucial for the fabrication of highly selective and sensitive electrochemical sensors for the determination of phenolic compounds.

On the other hand, the energy crisis is a major challenge for the future society. Solar energy is one of the most efficient renewable and clean energy sources and can fulfill energy requirements in the future [49]. Solar energy can be converted to electrical energy by employing photovoltaic devices (also called solar cells). Although traditional solar cells based on silicon are commercialized, they suffer from high cost and complicated manufacturing processes [50]. Thus, it is of great importance that we develop low-cost and highly efficient solar cells. In this regard, dye-sensitized solar cells (DSSCs), perovskite solar cells, polymer solar cells, and organic solar cells have been developed [51,52,53]. Among them, perovskite solar cells have demonstrated excellent power conversion efficiency but have their limitations, such as the presence of toxic lead and poor stability in moisture [54]. Thus, DSSCs have received significant attention and exhibited excellent stability in atmospheric conditions. DSSCs are composed of various components such as transparent conductive oxide electrodes, electron transport materials, a visible light sensitizer (dye), an electrolyte, and a counter electrode [55]. Each component has a significant role, but the counter electrode (CE) plays a vital role due to the presence of highly catalytic and conductive platinum (Pt) CE materials. Although Pt is an excellent CE material, its application is restricted due to the rarity of Pt and its high cost. Thus, it is necessary to find alternatives Pt-free CE materials for the development of low-cost and Pt-free DSSCs. In this context, various CE materials such as metal oxides, polymers, and carbon-based materials have been used as Pt-free CEs, but their performance was poor compared to Pt-based DSSCs [56,57,58]. Previously published articles have revealed that MXenes possess good conductivity with reasonable electro-catalytic properties, which makes them a promising electrode material for electrochemical sensing and dye-sensitized solar cell applications [59,60,61]. MXene materials are attracting growing interest among researchers working in different fields [62,63,64,65,66]. MXenes also exist in single-layered and multi-layered structures. Single-layered MXenes are characterized by their monolayer and thin structure and show superior electronic conductivity [67]. Single-layered MXenes also have enhanced surface reactivity that may be responsible for facilitating electron transfer during redox and electro-catalytic reactions. The high surface area and active sites in the single-layered MXenes may also improve electro-catalytic properties, which are beneficial for electrochemical applications [68]. Despite excellent features and properties, single-layered MXenes suffer from their inherent instability in aqueous environments, which can be responsible for their degradation and limit their practical applications. In contrast, multi-layered MXenes may offer advantages in terms of structural robustness and mechanical stability, which are useful for the development of highly stable electrochemical devices [69]. The interactions between the interlayers of the multi-layered MXenes may also improve conductivity through electronic coupling. However, this may happen in the multi-layered MXenes at the expense of accessibility to active sites compared to the single-layered MXenes. Additionally, multi-layered MXenes may be more easily processed and integrated into composites. However, multi-layered MXenes exhibit poor electro-catalytic properties due to the limited surface area and fewer exposed active sites compared to the single-layered MXenes [70]. Thus, it is clear that choice of single-layered or multi-layered MXenes for electro-catalysis may depend on the specific application requirements. The single-layered MXenes favor applications demanding high reactivity, whereas multi-layered MXenes are suitable for applications where stability and structural integrity are prioritized. MXenes can be distinguished as delaminated and non-delaminated MXenes. Delaminated MXenes are exfoliated into individual or a few layered sheets. The delaminated MXenes may have a high surface area, higher electrochemical activity, electrical conductivity, and better dispersion in polar solvents. Unfortunately, delaminated MXenes may face stability issues for long term application [71]. Although the delamination process produces high-quality MXenes, it is challenging to scale up for industrial applications. In contrast, non-delaminated MXenes are also known as multi-layered MXenes, which consist of stacked MXene sheets that are exfoliated from the MAX phase. The non-delaminated MXenes may have higher mechanical stability compared to the delaminated MXenes [72]. Delaminated and non-delaminated MXenes each have their own distinct advantages and disadvantages, making them suitable for various applications. As discussed above, delaminated MXenes can be characterized by their high surface area, superior dispersibility, and increased electrochemical/chemical reactivity and are well-suited for applications that demand rapid electron transport, high capacitance, and surface functionalization. Different synthesis methods, particularly etching processes, control the formation of multi-layered or delaminated MXenes. The direct HF etching method tends to yield multi-layered MXenes, whereas selective etching combined with intercalation agents such as dimethyl sulfoxide (DMSO) facilitates delamination into single or few layers. In multi-layered MXenes, the sheets remain stacked with weak van der Waals forces holding them together. However, intercalants such as Li^+^ ions weaken these forces and promote the exfoliation process into delaminated layers, which possess a higher surface area and reactivity. The properties of multi-layered and delaminated MXenes differ significantly. Multilayered MXenes exhibit higher mechanical stability but low surface area, which limits their active sites in sensing applications. Delaminated MXenes, with their increased surface area and better ion accessibility, demonstrate superior electrical conductivity and faster response times in electrochemical sensors. In addition, delaminated MXenes are preferred for energy storage devices due to enhanced ion intercalation capabilities, whereas multi-layered MXenes find use in structural reinforcement applications where mechanical properties are critical. In contrast, non-delaminated MXenes offer improved mechanical strength, which makes them an appropriate material for photovoltaic devices such as DSSCs. The selection of delaminated or non-delaminated MXenes may depend on the specific needs of the application. Previously, numerous reports were published on the use of single and multi-layered MXenes and their composites for the construction of electrochemical sensors and DSSCs. It is known that DSSCs and electrochemical sensors used semi-conducting metal oxides, MXenes, and other materials as catalysts. The role of the electro-catalyst is to catalyze the redox reactions and improve the charge transport process. Previously, it has been observed that MXenes have been widely used in both applications. We believe that merging these two applications in this mini-review article may be beneficial to understand the state of the art for the researchers in their early careers or starting to work on the fabrication of DSSCs or electrochemical sensors using MXene-based catalysts. 

Herein, we reported the mini-review article, which summarizes the previous reports on the use of MXenes and its composite materials for the development of phenolic sensors and DSSCs. The main objective of this review article is to summarize the recent reports on the synthesis of MXene-based composite materials towards the construction of phenolic sensors and Pt-free DSSCs. 

## 2. Synthesis Method for MXenes 

In the present scenario, various synthetic methods have been proposed for the preparation of MXenes. In this section, we have discussed the widely used synthetic procedures towards the preparation of MXenes.

### 2.1. In Situ HF Etching Method 

Recently, in situ HF etching methods have been widely used for the preparation of MXenes, which involve the use of lithium fluoride (LiF)/hydrochloric acid (HCl) or LiF/sodium fluoride (NaF). The mechanism can be explained as given below [36].

Ti_3_AlC_2_ + 3HCl + 3 LiF = Ti_3_C_2_ + 3LiCl + AlF_3_ +1.5 H_2_ (1)

Ti_3_C_2_ + 2HF = Ti_3_C_2_(F)_2_ + H_2_ (2)

Ti_3_C_2_ + 2HCl = Ti_3_C_2_(Cl)_2_ + H_2_ (3)

Ti_3_C_2_ + 2H_2_O = Ti_3_C_2_(OH)_2_ + H_2_ (4)

Ti_3_C_2_ + 2H_2_O = Ti_3_C_2_(O)_2_ + 2H_2_ (5)

Reported literature suggested that the in situ HF etching method may improve the quality of the MXene and MXene and do not need sonication treatment for the delamination of MXenes [73]. Although the in situ method requires low energy but has some limitations, such as a time-consuming dry process [74,75].

### 2.2. Direct HF Etching Method 

Naguib et al. [8] reported a novel direct HF etching method for the preparation of MXenes. HF is a very strong acid that needs significant precautions before use. The strong HF assists the etching process to transform the MAX phase to MXene by eliminating Al layers. The byproducts such as AlF_3_ and H_2_O may further react to generate –OH, -F, or =O terminations. The mechanism has been described below.

Ti_3_AlC_2_ + 3HCl + 6 LiF = 2Ti_3_C_2_ + 2AlF_3_ +3H_2_ (6)

Ti_3_C_2_ + 2HF = Ti_3_C_2_(F)_2_ + H_2_ (7)

Ti_3_C_2_ + 2H_2_O = Ti_3_C_2_(OH)_2_ + H_2_ (8)

Ti_3_C_2_ + 2H_2_O = Ti_3_C_2_(O)_2_ + 2H_2_ (9)

The direct HF etching method may be more efficient to prepare the MXenes compared to the in situ etching method. However, HF is extremely corrosive and toxic in nature, which limited its application. 

### 2.3. Hydrothermal Method

Li et al. [76] proposed the hydrothermal-assisted synthesis of MXenes. In this method, deionized (DI) water was initially boiled and kept in an argon (Ar) gas atmosphere for 0.5 h. Further, sodium hydroxide (NaOH) was added to the water. Further, 100 mg of Ti_3_AlC_2_ powder was added to the 25 mL of NaOH solution and poured into a 50 mL autoclave. The autoclave was tightened and heated at 65 °C for 12 h. After cooling down the autoclave, the MXene sample was collected and filtered with a membrane (PVDF; 0.1 µm in pore size), and the obtained product was found to be MXene. Peng et al. [77] reported hydrothermal synthesis of MXene. The mechanism can be described as follows:

Ti_3_AlC_2_ + OH^−^ + 5H_2_O = Ti_3_C_2_(OH)_2_ + Al(OH)_4_^−^ + 2.5 H_2_ (10)

Ti_3_AlC_2_ + OH^−^ + 5H_2_O = Ti_3_C_2_(O)_2_ + Al(OH)_4_^−^ + 3.5 H_2_ (11)

### 2.4. Exfoliation Method

The exfoliation method has various advantages for the preparation of layered materials [72]. Common intercalants such as tetrabutylammonium hydroxide (TBAOH), dimethyl sulfoxide (DMSO), hydrazine, urea, and NH_4_^+^ can be used to generate these spaces [78,79,80,81,82]. Chia and coworkers utilized TBAOH as an intercalant to exfoliate the Ti_3_C_2_T_x_ MXene [83]. The authors found that few layered MXenes are obtained on the use of TBAOH, whereas multilayered MXenes are formed by employing NH_4_HF_2_. 

### 2.5. Electrochemical Etching Method

Yang et al. [84] reported the electrochemical synthesis of MXene by tailoring the composition of the electrolyte. Li et al. [85] reported the synthesis of V_2_CT_x_ MXene by using the electrochemical etching method. Since each method has its own advantages and disadvantages. HF treatment has been widely used for the preparation of high-quality MXenes. The major advantages of this method include the high yield of MXenes, but it has limitations such as highly toxic HF, which needs careful handling. HF treatment is dangerous in terms of safety and needs precautions before its handling. It is advisable for the researchers that they should not deal with HF in the laboratories when they are alone. In this regard, an in situ HF etching method has been developed, which is a relatively safer method compared to the direct HF treatment. However, this method still involves HF and needs cautious handling during the reaction and washing process. The major challenge for this method is lower etching efficiency compared to the direct HF treatment. In addition, this method was found to be effective for Ti_3_C_2_T_x_ MXenes only. The hydrothermal method can be beneficial to control the morphology of the MXene by optimizing the effects of pH, temperature, and pressure. However, the hydrothermal method may have some limitations, such as the generation of high pressure in the autoclave during synthesis time, which raised safety concerns. The hydrothermal method may also introduce defects or incomplete etching of the MAX phase, which can affect their performance and activity. On the other hand, the exfoliation method has several advantages, such as simplicity and high yield [86]. However, this method also has many limitations and challenges, such as limited control over the surface chemistry, such as the presence of −OH, −O, and -F functional groups, and inconsistent product quality. The electrochemical method was also adopted for the preparation of MXenes, and it was considered an environmentally friendly and fluorine-free method for the synthesis of MXenes. The electrochemical method allows precise control over the degree of etching and may introduce additional surface modifications during the synthesis of MXenes. However, the electrochemical method is slower compared to the direct HF treatment method. It can be used only for selected MAX phases, and the etching process is less uniform compared to the direct HF treatment. Thus, it can be concluded that direct HF treatment is the more efficient approach for the preparation of MXenes with good quality compared to the other methods. It is also known that single-layered MXenes possess high electro-catalytic properties but lower stability compared to the multi-layered MXenes. It is believed that electrochemical sensing devices need highly catalytic materials. The electrochemical devices can significantly detect analytes rapidly and are used for a short periods of time. Thus, single or a few layered MXenes would be suitable for the construction of electrochemical sensors. On the other hand, DSSCs are the photovoltaic devices that may be used for a long time with continuous exposure to sunlight. Thus, electrode materials should have high stability along with the catalytic properties. We believe that multi-layered MXenes and their composites may be a good choice for the development of Pt-free counter electrodes for DSSCs application. It may be noted that a novel method is required, which may overcome the limitations and challenges of the present synthetic procedures. However, in the present scenario, researchers are widely using direct HF treatment-assisted methods for the preparation of high-quality MXenes and further exploring sonochemical approaches to obtain the hybrid composite materials. For sensing and DSSC applications, electrode materials are dispersed in a suitable solvent and subsequently coated on to the surface of the electrodes. High-quality MXenes can be prepared by using direct HF treatment. Thus, we believe that the direct HF etching treatment-assisted sonochemical method may be used for the synthesis of MXene-based hybrid composite materials for sensing and DSSC applications. 

## 3. Progress in MXene-Based Electrode Materials for Phenolic Compound Sensing

Previously, MXenes and their composite materials have been synthesized by using various methods and explored as catalysts for electrochemical sensing applications. The desirable features of the electro-catalysts are high surface area, electrical conductivity, and nanostructured morphology, which can significantly affect the performance of the electrochemical sensors. Published literature showed that HF-based etching method has been widely adopted for the preparation of MXenes, while the ultra-sonication method is used for the preparation of MXene-based hybrid composite materials. The properties and synergistic interactions can be tuned by varying the weight or mole percentage of the MXenes to other supporting materials during the ultra-sonication method. This may improve the performance of the electrochemical sensors. In this section, we have compiled published articles on the construction of electrochemical sensors towards the determination of phenolic compounds.

### 3.1. Bisphenol A Sensor

**Figure 1:**
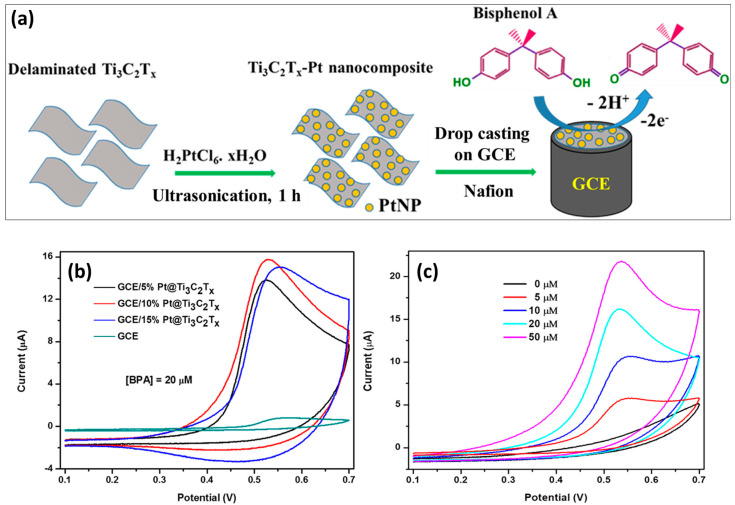
(**a**) Schematic representation of the synthesis of Ti_3_C_2_T_x_-Pt and surface modification of GCE. (**b**) CV responses of the different electrodes in 20 µM BPA at scan rate of 100 mV/s. (**c**) CV curves of the 10%Pt@Ti_3_C_2_T_x_/GCE in different concentrations of BPA at a scan rate of 100 mV/s. Reprinted with permission [87].

Bisphenol A (BPA) is one of the most widely used materials for various applications, such as epoxy resins and plastic items. However, the toxicity of BPA may disrupt the endocrine system. Thus, it is important to develop the electrochemical sensor for the sensing of BPA. Rasheed et al. [87] reported the fabrication of a novel catalyst for the sensing of BPA. The authors synthesized the Pt@Ti_3_C_2_T_x_ composite using novel synthetic protocols, as demonstrated in Figure 1a. The synthesized Pt@Ti_3_C_2_T_x_ composite was coated on the surface of the GCE using a drop casting approach. The authors also optimized the synergistic effects by tuning the percentage of the Pt content. The CV curves of the Pt@Ti_3_C_2_T_x_/GCE with different Pt percentages for the determination of BPA are shown in Figure 1b. It was reported that 10% Pt@Ti_3_C_2_T_x_/GCE has the highest current response for the oxidation of BPA compared to the other electrodes. Furthermore, authors investigated the effect of different concentrations on the performance of the 10% Pt@Ti_3_C_2_T_x_/GCE (Figure 1c). The current response linearly increases with increasing concentrations of the BPA. The 10%Pt@Ti_3_C_2_T_x_/GCE exhibited a limit of detection (LOD) of 32 nM with excellent selectivity, repeatability, fast response, and decent stability. The authors also reported that 10% Pt@Ti_3_C_2_T_x_/GCE has excellent BPA recoveries in drinking water and milk samples towards real sample applications. The improved electrochemical performance may be attributed to the presence of synergistic interactions between Pt and Ti_3_C_2_T_x_.

Previously, Rajendran et al. [88] synthesized a hybrid composite of graphene/Ti_3_C_2_T_x_ composite using a top-down approach. The synthesized graphene/Ti_3_C_2_T_x_ was coated on the GCE, and its performance was checked in the presence of the [Fe(CN)_6_]^3−/4−^ system using CV. The authors found that graphene/Ti_3_C_2_T_x_/GCE had excellent catalytic properties compared to the other electrodes. Furthermore, the electrochemical performance of the graphene/Ti_3_C_2_T_x_/GCE was determined by employing differential pulse voltammetry (DPV) and amperometry methods. Graphene/Ti_3_C_2_T_x_/GCE demonstrated decent LOD of 0.35 µM and 4.08 nM using DPV and amperometry, respectively, for the sensing of BPA. The authors also found that graphene/Ti_3_C_2_T_x_/GCE has excellent stability and selectivity for the determination of BPA. The real sample studied also showed that BPA can be recovered in the range of 99.2–104.5% using the spike method. Thukkaram et al. [89] prepared MXene/vanadium pentoxide (Ti_3_C_2_T_x_/V_2_O_5_) composites for the determination of BPA using electrochemical methods. The Ti_3_C_2_T_x_/V_2_O_5_ modified electrode demonstrated an LOD of 87 nM with a wide dynamic linear range of 411–31.2 µM. Qu et al. [90] also proposed the fabrication of novel electrochemical sensors for the monitoring of BPA. Authors prepared Pt nanoparticles (NPs) modified single-walled carbon nanotubes (Pt@SWCNTs)/Ti_3_C_2_/graphene oxide (GO). The synthesized Pt@SWCNTs/Ti_3_C_2_/GO was coated on a screen-printed carbon electrode (SPCE) using a simple fabrication process. The modified SPCE demonstrated excellent stability, reproducibility, and other acceptable results.

### 3.2. 4-Nitrophenols Sensors

In the past few years, sensing of 4-nitrophenol (4-NP) has received significant attention because of the toxicity of 4-NP. 4-NP and its derivatives, such as 2-NP or 3-NP, are used in dyes, chemicals, pesticides, explosives, and pharmaceutical industries. 4-NP and their derivatives are also known as toxic and carcinogenic compounds, which need immediate attention. The US Environmental Protection Agency (EPA) also mentioned 4-NP as a hazardous pollutant. Thus, it is crucial to monitor the level of 4-NP in the environment. Krishnamoorthy et al. [91] prepared a stacked layer of chemically exfoliated Ti_3_C_2_T_x_ MXene for the construction of the 4-NP sensor. The Ti_3_C_2_T_x_ MXene was coated on the GCE, and its performance was studied using CV and DPV methods. The Ti_3_C_2_T_x_ MXene-coated GCE was capable of the efficient determination of 4-NP using the DPV method. The Ti_3_C_2_T_x_ MXene/GCE exhibited the LOD of 42 nM/L with a linear dynamic range of 0.5 µM to 100 µM. Ti_3_C_2_T_x_ MXene/GCE exhibited a good selective nature for the detection of 4-NP in the presence of various interfering compounds. The real sample recoveries of 95–99% were also found in real-time applications with decent repeatability and reproducibility. The sensing mechanism for the detection of 4-NP is expressed as follows:

Ti_3_C_2_T_x_ + 4e− = (Ti_3_C_2_T_x_)^4−^ (12)

(Ti_3_C_2_T_x_)^4−^ + 4-NP + 4H^+^ = Ti_3_C_2_T_x_ + 4-HAP + H_2_O (13)

**Figure 2:**
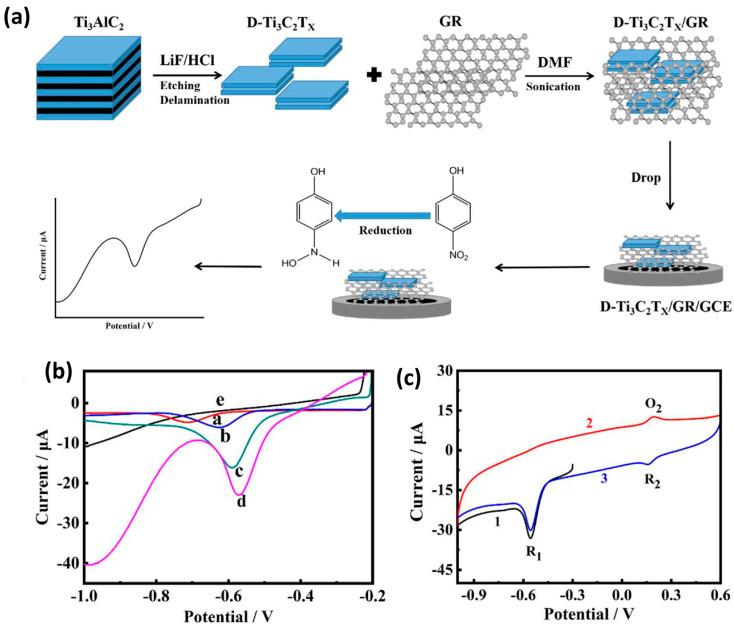
(**a**) Schematic diagram shows the synthesis of D-Ti_3_C_2_T_x_/GR and electrode modification for 4-NP sensing. (**b**) DPV curves of the bare GCE (curve a) and different modified electrodes D-Ti_3_C_2_T_x_/GCE (curve b), GR/GCE (curve c), D-Ti_3_C_2_T_x_/GR/GCE (curve d) for 50 µM 4-NP, and D-Ti_3_C_2_T_x_/GR/GCE (curve e) in the absence of 4-NP. (**c**) CV curve of D-Ti_3_C_2_T_x_/GR/GCE for 50 µM 4-NP. Reprinted with permission [92].

In another report, Wang et al. [92] proposed the synthesis of delaminated titanium carbide (D-Ti_3_C_2_T_x_) MXene using the minimally intensive layer delamination (MILD) method followed by the self-assembly approach to form the D-Ti_3_C_2_T_x_/GR composite (Figure 2a). 

The D-Ti_3_C_2_T_x_/GR composite was coated on the active surface area of the GCE for the sensing of 4-NP. Electrochemical performance of the D-Ti_3_C_2_T_x_/GR composite modified electrode was evaluated using CV and DPV methods. DPV curves of the bare GCE and different modified GCE were recorded in the presence of 4-NP. The D-Ti_3_C_2_T_x_/GR composite modified GCE demonstrated higher electro-catalytic activity for the detection of 4-NP compared to the bare GCE and other modified electrodes (Figure 2b). The D-Ti_3_C_2_T_x_/GR/GCE shows no redox peaks in the absence of 4-NP, whereas the presence of 4-NP demonstrated oxidation/reduction peaks (Figure 2b). CV results demonstrated that sensing of 4-NP involves oxidation (O_2_) and reduction (R_1_ and R_2_) peaks (Figure 2c). The D-Ti_3_C_2_T_x_/GR/GCE showed LOD of 0.16 µM with a linear range of 1 to 175 µM. The D-Ti_3_C_2_T_x_/GR/GCE has excellent selectivity, stability, reproducibility, and recovery in real sample analysis towards the detection of 4-NP in environmental samples. Lei et al. [93] reported the preparation of hydrothermally assisted synthesis of exfoliated multi-layered oxidized Ti_3_C_2_T_x_ MXene for the construction of a 4-NP sensor. The oxidized Ti_3_C_2_T_x_ MXene was coated on the GCE surface towards the detection of 4-NP using the DPV method. A decent LOD of 0.11 µM and a linear range of 0.5 to 25 µM were achieved for the determination of 4-NP using an oxidized Ti_3_C_2_T_x_ MXene-modified electrode. The proposed sensor also demonstrated excellent selectivity towards the sensing of 4-NP in the presence of various interfering species (Pb^2+^, catalase (CAT), Cd^2+^, tri-nitrophenol, and phenol). The authors also proposed excellent recovery in a waste water real-time sample. In 2024, Gopi et al. [94] reported the preparation of silver bismuth sulfide (AgBiS_2_)/MXene composite and used it as an electrode modifier for the determination of 4-NP. The proposed electrode demonstrated excellent LOD of 0.00254 µM with a linear range of 0.02–1869 µM. 

### 3.3. Catechol/Hydroquinone Sensors

Catechol (CAT) has carcinogenic properties, as reported by the International Agency for Research on Cancer (IARC). It is important to monitor and control the amount of CAT in waste water to avoid its negative impacts on human health and the environment. In this regard, Chandran et al. [95] found that MXene may be a promising electrode material for the construction of CAT sensors due to its higher conductivity and robust stability. The authors reported the fabrication of a biosensor using laccase (Lac) immobilized Au/MXene modified GCE as a working electrode via the CV method. The authors used nafion as a binder to improve the adhesiveness of the electrode modifiers on the surface of GCE. The Lac/Au/MXene/GCE exhibited LOD of 0.05 µM with a linear range of 0.05 to 0.15 µM and robust stability. The Lac/Au/MXene/GCE also demonstrated acceptable selectivity for the detection of CAT in the presence of various interfering compounds. 

**Figure 3:**
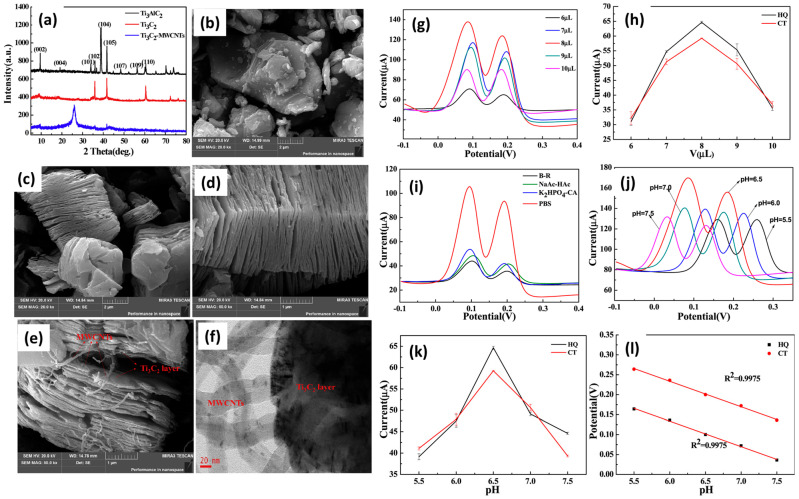
(**a**) XRD patterns of Ti_3_AlC_2_, Ti_3_C_2_, and Ti_3_C_2_/MWCNTs. SEM image of (**b**) Ti_3_AlC_2_, (**c**,**d**) Ti_3_C_2_, and (**e**) Ti_3_C_2_/MWCNTs. (**f**) TEM image of Ti_3_C_2_/MWCNTs composite. (**g**,**h**) Effect of loading amount (6, 7, 8, 9, and 10 µL) of Ti_3_C_2_-MWCNTs/GCE. (**i**) DPV responses of Ti_3_C_2_-MWCNTs/GCE in 100 µM CAT and HQ in different supporting electrolytes. (**j**) DPV responses of the Ti_3_C_2_-MWCNTs/GCE 100 µM CAT and HQ in different pH conditions. (**k**) pH–current curves and (**l**) linear graph of pH versus peak potential. Reprinted with permission [96].

Multi-walled carbon nanotubes (MWCNTs) are the carbon materials with excellent physicochemical properties. The combination of MWCNTs with MXene may enhance the catalytic properties of the composite materials. Huang et al. [96] synthesized a Ti_3_C_2_/MWCNTs composite using novel synthetic strategies. The powder X-ray diffraction (XRD) patterns of the Ti_3_AlC_2_, Ti_3_C_2_, and Ti_3_C_2_/MWCNTs are demonstrated in Figure 3a. It is clear that Ti_3_C_2_ and Ti_3_C_2_/MWCNTs were successfully formed. The scanning electron microscopy (SEM) image of Ti_3_AlC_2_ showed the presence of flake-like surface morphology, as shown in Figure 3b. The reported SEM images of the Ti_3_C_2_ have been demonstrated in Figure 3c,d, which suggested the presence of an exfoliated, layered structure of the prepared MXene. Figure 3e clearly revealed the presence of MWCNTs on the surface of the Ti_3_C_2_ MXene and confirmed the formation of the Ti_3_C_2_/MWCNTs composite. The transmission electron microscopy (TEM) also confirmed the formation of the Ti_3_C_2_/MWCNTs composite (Figure 3f).

The prepared Ti_3_C_2_/MWCNTs composite was deposited on the GCE surface, and the loading amount was optimized towards the determination of CAT and hydroquinone (HQ) simultaneously. Authors found that 8 µL loaded Ti_3_C_2_/MWCNTs composite on GCE surface has the highest catalytic activity for the determination of CAT and HQ (Figure 3g,h). The authors also optimized different electrolytes to improve the sensing activity of the Ti_3_C_2_/MWCNTs composite modified electrode. The authors reported that the Ti_3_C_2_/MWCNTs composite modified electrode has higher electro-catalytic activity in PBS solution (Figure 3i). The pH of the PBS solution was also optimized as 6.5 for the improved sensing of HQ and CAT using a Ti_3_C_2_/MWCNTs composite modified electrode (Figure 3j). The pH-versus-current curves and pH versus peak potential graphs are shown in Figure 3k,l, respectively. The Ti_3_C_2_/MWCNTs composite modified electrode showed an LOD of 3.9 nM for the sensing of CAT with a linear range of 2 to 150 µM. Authors also reported excellent stability, repeatability, and selectivity for the proposed sensor with decent recoveries in real samples. Ranjith et al. [97] fabricated one-dimensional (1D) MnMoO_4_ nanofibers coupled with a few layered exfoliated 2D MXene. The synthesized MnMoO_4_/MXene composite was explored as the catalyst material to enhance the sensing of the modified electrode towards the determination of CAT and HQ. The MnMoO_4_/MXene composite/GCE demonstrated excellent performance for the detection of CAT and HQ with remarkably good LODs of 0.30 µM and 0.26 µM, respectively. Huang et al. [98] reported novel hetero-structure of MXene/metal–organic frameworks (MOFs) for the sensing of Cat and HQ. The proposed electrode material (Ti_3_C_2_/MOF) was prepared by combining MOF-NH_2_-derived nitrogen-doped porous carbon with alk-Ti_3_C_2_. The proposed material was applied as an electro-catalyst for the construction of CAT and HQ sensors, which demonstrated acceptable sensing performance in terms of LOD and sensitivity. The LOD of 4.8 nM and 3.1 nM were obtained for HQ and CAT, respectively, with a linear range of 0.5 to 150 µM. Zhang et al. [99] proposed a novel material for combining FeCu-MOF-919/Ti_3_C_2_T_x_ composites for the detection of resorcinol (Res). The prepared FeCu-MOF-919/Ti_3_C_2_T_x_ composite was applied on the surface of the electrode. The FeCu-MOF-919/Ti_3_C_2_T_x_ composite modified electrode exhibited excellent LOD of 0.08 µM with wide linear range of 0.5 to 152.5 µM and outstanding stability. The authors also applied the proposed sensor for real-time applications, which demonstrated acceptable recoveries in the range of 96.25 to 103.37% with a decent RSD of 2.18%. Lu et al. [100] also reported a novel electrode material for the sensing of CAT using the electrochemical method. The authors prepared amino-functionalized bimetallic organic framework materials (Fe@Ti-MOF-NH_2_) and coupled them with Ti_3_C_2_ MXene, and the synthesized material was labeled as MXene/Fe@Ti-MOF-NH_2_. Furthermore, a novel electrode (MIP/pTHi/MXene/Fe@Ti-MOF-NH_2_/GCE) was fabricated for the electrochemical determination of CAT. The authors employed square wave voltammetry (SWV) for the determination of CAT. Initially, electro-catalytic properties of the MIP/pTHi/MXene/Fe@Ti-MOF-NH_2_/GCE were checked using CV and EIS methods. In further investigations, MIP/pTHi/MXene/Fe@Ti-MOF-NH_2_/GCE was applied as a CAT sensor, and its performance was evaluated using the SWV method. The MIP/pTHi/MXene/Fe@Ti-MOF-NH_2_/GCE demonstrated LOD of 0.54 µM with wide dynamic linear range of 1 to 4000 µM. The proposed CAT sensor also showed excellent selectivity, reproducibility, acceptable stability, and repeatability for detecting CAT. Additionally, the proposed sensing system demonstrates strong specific recognition when analyzing environmental matrices and biological fluids in real samples, yielding satisfactory results. Consequently, this signal-enhanced ratio-metric MIP electrochemical sensing approach can accurately and selectively detect and analyze various other substances. The electrochemical performance of the reported electrochemicals has been compiled in Table 1. It is well known that conventional materials such as graphene and metal oxides demonstrated decent performance for the fabrication of electrochemical sensors towards the determination of phenolic compounds. In this connection, Wang et al. reported an interesting LOD of 1.9 nM using MOF/graphene composite [101]. A decent LOD of 33 nM was reported for the fabrication of CC using graphene/Sn MOF composite [102]. Zaidi et al. [103] achieved the LOD of 10 nM using manganese dioxide (MnO_2_) nanorods. Arpitha et al. [104] also reported the fabrication of a zinc oxide/cobalt oxide composite for the sensing of resorcinol, which exhibited a decent LOD value of 3.226 µM and 2.92 µM, respectively. On the other side, MXene-based materials demonstrated improved LOD compared to the conventional materials, as shown in Table 1. The MXene-based sensors possess excellent catalytic properties, which improved the sensing mechanism for the determination of phenolic compounds.

**Table 1:** Comparison of the electrochemical performance of the MXenes and their composite-based electrochemical sensors.

Material	Etching Agent or Method	Layered Structure	Sensing Analyte	LOD (µM)	Linear Range (µM)	Real Sample Analysis	Sensing Technique	References
10%Pt@Ti_3_C_2_T_x_/GCE	LiF/HCl	Multi-layered	BPA	0.032	0.05 to 5.0	Fresh milk	DPV	[87]
Gr/MXene/GCE	HF	Multi-layered	BPA	0.004	0.01 to 0.18	Food package, drinking cup, and feeding bottle	Amperometry	[88]
Gr/MXene/GCE	HF	Multi-layered	BPA	0.35	1 to 10		DPV	[88]
V_2_O_5_@Ti_3_C_2_T_x_	HF	Multi-layered	BPA	0.087	0.0041 to 31.2	-	DPV	[89]
Pt@SWCNTs-Ti_3_C_2_-rGO/SPCE	LiF/HCl	Multi-layered	BPA	0.0028	0.006 to 9.8	Thermal paper	DPV	[90]
Ti_3_C_2_T_x_	HF	Multi-layered	4-NP	0.042	0.5 to 25	Tap water	DPV	[91]
D-Ti_3_C_2_T_x_/GR	LiF/HCl	Multi-layered	4-NP	0.16	1 to 175	Sea water and Tap water	DPV	[92]
Ti_3_C_2_T_x_	NaF/HCl	Multi-layered	4-NP	0.11	0.5 to 25	Waste water	DPV	[93]
Mxene-AgBiS_2_/GCE	HF	Multi-layered	4-NP	0.0025	10 to 78	Tap water	DPV	[94]
Lac/Au/MXene/GCE	HF	Multi-layered	CC	0.05	0.05 to 0.15	-	CV	[95]
Ti_3_C_2_-MWCNT	LiF/HCl	Multi-layered	CC	0.0039	2 to 150	Waste water	DPV	[96]
Ti_3_C_2_-MWCNT	LiF/HCl	Multi-layered	HQ	0.0066	2 to 150	Waste water	DPV	[96]
Alk-Ti_3_C_2_/N-PC	HF	Multi-layered	HQ	0.0048	0.5 to 150	Industrial waste water	DPV	[98]
Alk-Ti_3_C_2_/N-PC	HF	Multi-layered	CC	0.0031	0.5 to 150	Industrial waste water	DPV	[98]
FeCu-MOF-919/Ti_3_C_2_T_x_/GCE	LiF/HCl	-	RS	0.08	0.5 to 152.5	Tap water	DPV	[99]
MIP/pTHi/MXene/Fe@Ti-MOF-NH_2_/GCE	HF	Multi-layered	CC	0.54	1 to 4000	Tap water and human urine	SWV	[100]

## 4. Progress in DSSCs

The CE plays an important role in the fabrication of cost-effective DSSCs. It is one of the most expensive components in the structure of DSSCs due to the high cost of Pt. It is required to develop the Pt-free DSSCs to reduce the cost of Pt-based DSSCs. Herein, we have compiled the progress in the fabrication of Pt-free CE-based DSSCs using MXene or MXene-based composites as CE materials. 

### 4.1. Pristine MXenes as CE

2D materials with high conductivity are the desirable CE materials for DSSC applications. Developing highly stable and efficient Pt-free CEs is a significant challenge for DSSC applications. Ti₃C₂Tₓ MXene, known for its excellent catalytic activity and conductivity, has attracted interest as a CE material in DSSCs. MXenes are the promising candidate for their application in DSSCs due to their excellent conductivity and robust stability. In this context, 2D-layered Ti_3_C_2_ MXene was prepared using a low-temperature processed etching method [105]. The authors used the synthesized Ti_3_C_2_ MXene as CE material in the development of the Pt-free DSSCs. The constructed DSSCs using Ti_3_C_2_ MXene CE exhibited an interesting PCE of 9.57%, which may be attributed to the superior charge transfer/mass transport properties and 2D layer structure of Ti_3_C_2_ MXene [105]. 

**Figure 4:**
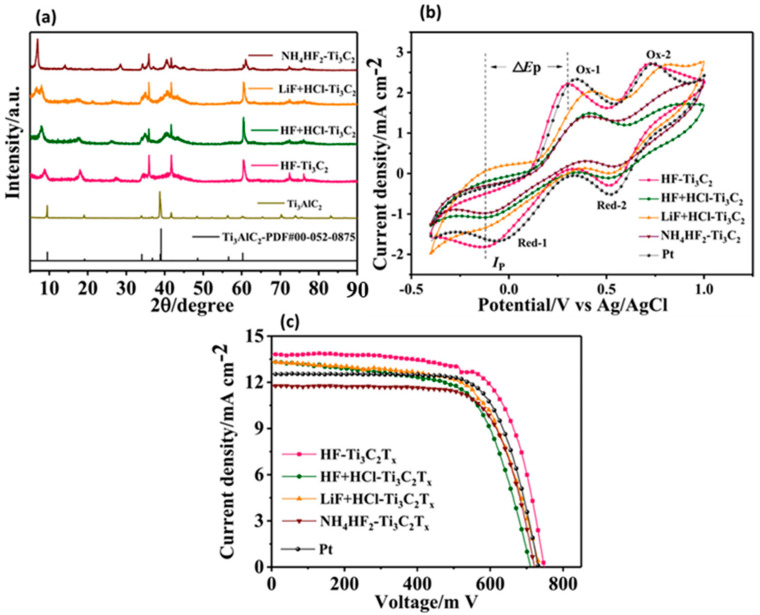
(**a**) XRD results for Ti_3_AlC_2_ and different laminated Ti_3_C_2_T_x_ MXenes. (**b**) CV curves of the different laminated Ti_3_C_2_T_x_ MXenes in redox systems. (**c**) J–V curves of the different laminated Ti_3_C_2_T_x_ MXene CE-based DSSCs. Reprinted with permission [108].

Xu et al. [106] also proposed the fabrication of MXene-based CEs for DSSC application. Authors synthesized two types of MXenes with different metal contents. In this connection, Ti_3_C_2_ and vanadium carbide (V_2_C) were prepared via etching method. The synthesized MXenes were applied for the fabrication of Pt-free DSSC and V_2_C MXene-based DSSCs exhibited a PCE of 4%. In contrast, T_i3_C_2_ MXene CE-based DSSCs demonstrated improved PCE of 5.3%, which is relatively higher than that of the V_2_C MXene CE-based DSSCs. The optimized results demonstrated an acceptable PCE of 7.1%. The excellent performance of the DSSCs may be ascribed to the prominent mass transport and charge transport properties of the Ti_3_C_2_ MXene, which is due to its 2D structure. In another study, Ahmad et al. [107] fabricated DSSCs using Ti_3_C_2_ MXene as Pt-free photovoltaic devices, which demonstrated excellent performance in terms of PCE of 8.68%. Nie et al. [108] adopted novel strategies to improve the photovoltaic performance of the Ti_3_C_2_T_x_ MXene-modified CE-based DSSCs. The authors prepared different laminated Ti_3_C_2_T_x_ MXenes, and their XRD patterns are shown in Figure 4a. The observations confirmed the formation of laminated Ti_3_C_2_T_x_ MXenes. The electro-catalytic performance of the different laminated Ti_3_C_2_T_x_ MXene-based CEs was checked by obtaining their CV curves in a redox system. The obtained results are demonstrated in Figure 4b. It was reported that HF-Ti_3_C_2_T_x_ CE exhibited higher catalytic activities compared to the other CEs. The catalytic properties of the HF-Ti_3_C_2_T_x_ CE were also comparable with Pt CE, as shown in Figure 4b. This may be ascribed to the higher specific surface area (13.799 cm^2^/g) of the HF-Ti_3_C_2_T_x_ compared to the other laminated MXene samples. In further studies, authors fabricated DSSCs using different laminated Ti_3_C_2_T_x_ MXenes CE-based DSSCs. The photocurrent density–voltage (J–V) curves of the different laminated Ti_3_C_2_T_x_ MXenes and Pt CE-based DSSCs are shown in Figure 4c. The observations showed that HF-Ti_3_C_2_T_x_ CE-based DSSCs exhibited the highest PCE of 7.15% with open circuit voltage (Voc), photocurrent density (Jsc), and fill factor (FF) of 0.749 V, 13.75 mA/cm^2^, and 0.71, respectively. In contrast, other CE-based devices exhibited lower performance compared to the HF-Ti_3_C_2_T_x_ CE based DSSCs. Even the authors found that a Pt CE-based device has a lower PCE of 6.57%. Therefore, authors proposed that HF-Ti_3_C_2_T_x_ is promising CE material for Pt-free DSSC applications. 

### 4.2. MXene-Based Composite Materials as CE Materials

**Figure 5:**
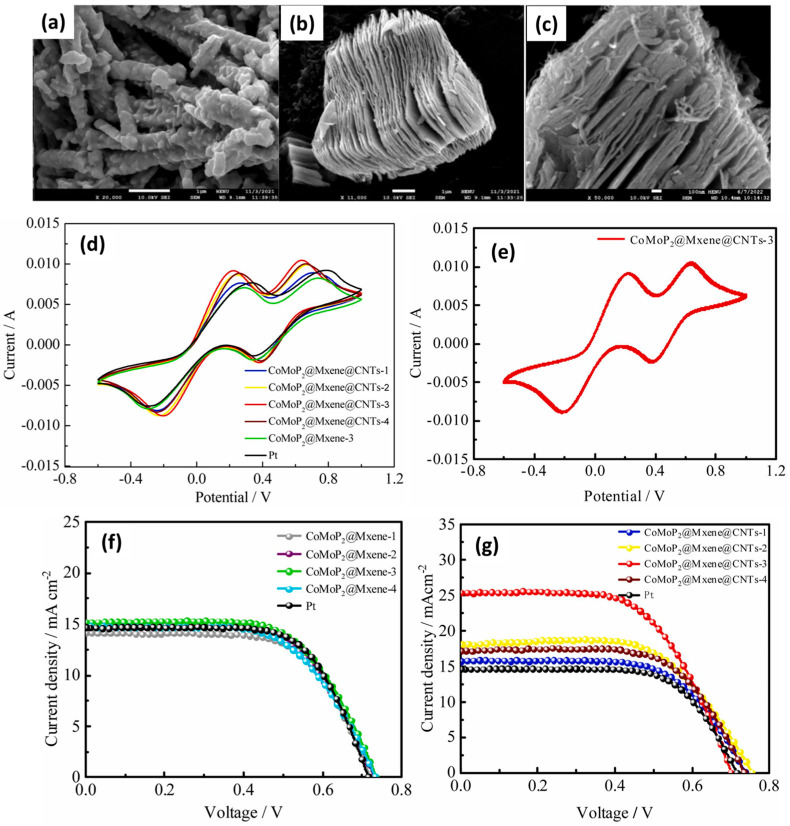
SEM image of (**a**) CoMoP_2_, (**b**) Ti_3_C_2_T_x_, and (**c**) CoMoP_2_@Mxene@CNTs-3. (**d**) CV curves of different CEs in the redox system. (**e**) Thirty CV curves of CoMoP_2_@Mxene@CNTs-3 in redox system. (**f**) J–V curves of CoMoP_2_@Mxene-1, CoMoP_2_@Mxene-2, CoMoP_2_@Mxene-3, and CoMoP_2_@Mxene-4. (**g**) J–V curves of CoMoP_2_@Mxene@CNTs-1, CoMoP_2_@Mxene@CNTs-2, CoMoP_2_@Mxene@CNTs-3, and CoMoP_2_@Mxene@CNTs-4. Reprinted with permission [110].

In the past few years, various CE materials were adopted as Pt-free electrode materials for the construction of DSSCs. It is believed that composite materials may exhibit better properties and performance for the development of Pt-free DSSCs. Di et al. [109] proposed a synthesis of novel CE material to enhance the cost-effectiveness and photovoltaic performance of the Pt-free DSSCs. The authors prepared Ni species over the surface of the plasmonic titanium nitride (TiN@Ni) by employing the wetness impregnation method. In further investigations, the prepared TiN@Ni was combined with MXene for the construction of Pt-free CE towards the development of DSSCs. The TiN@Ni-MXene CE-based DSSCs showed an interesting PCE of 8.08%, which is relatively higher than that of the Pt CE (7.59%)-based DSSCs. It is interesting that PCE of the TiN@Ni-MXene CE-based DSSCs is remarkable compared to the Pt-based DSSCs. This offers the potential of MXene based materials for the fabrication of cost-effective and Pt-free DSSCs. It is reported that cobalt molybdenum phosphide (CoMoP_2_) has excellent electro-catalytic properties, and it would be of great significance to combine it with highly conducting MXene materials to further improve their electro-catalytic activities for the regeneration of redox electrolytes. Therefore, He et al. [110] reported the synthesis of CoMoP_2_, Ti_3_C_2_T_x_, and CoMoP_2_@Mxene@CNTs-3 using simple approaches. The SEM picture of the CoMoP_2_ revealed the presence of aggregated short rod-like structure, and the rough surface of the short rods was embedded in many blocks and generated more active sites (Figure 5a). The SEM results for Ti_3_C_2_T_x_ showed the typical multi-layer structure of MXene (Figure 5b). The SEM results of the CoMoP_2_@Mxene@CNTs-3 exhibited the presence of CoMoP_2_ on the surface of Mxene@CNTs (Figure 5c). The authors designed and fabricated various combinations of the CE materials to improve their catalytic properties. The CV curves of the CoMoP_2_@Mxene-3, Pt, CoMoP_2_@Mxene@CNTs-1, CoMoP_2_@Mxene@CNTs-2, CoMoP_2_@Mxene@CNTs-3, and CoMoP_2_@Mxene@CNTs-4 CEs are demonstrated in Figure 5d, and it was observed that CoMoP_2_@Mxene@CNTs-3 CE has the highest electro-catalytic activities for the redox reactions. The stability of the CoMoP_2_@Mxene@CNTs-3 CE was also studied by recording 30 cycles (Figure 5e). The DSSCs were fabricated using Pt, J–V curves of CoMoP_2_@Mxene-1, CoMoP_2_@Mxene-2, CoMoP_2_@Mxene-3, and CoMoP_2_@Mxene-4 as CEs. 

**Figure 6:**
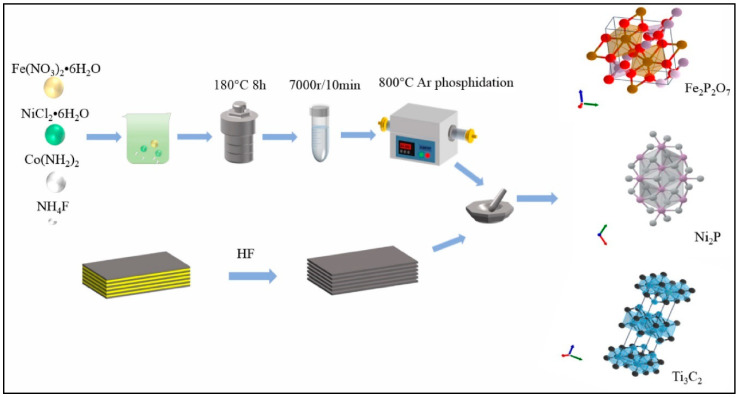
Schematic representation of the synthesis of the materials. Reprinted with permission [112].

The obtained results showed that CoMoP_2_@Mxene-3-based DSSCs have the highest PCE of 7.08% (Figure 5f). In the other case, CoMoP_2_@Mxene@CNTs-3-based DSSCs exhibited enhanced PCE of 10.64% with Jsc = 25.43 mA/cm^2^, Voc = 0.701, and FF = 0.59 (Figure 5g). This may be ascribed to the synergistic interactions in the prepared CoMoP_2_@Mxene@CNTs-3 material. Fan and coworkers [111] reported the synthesis of novel CE materials (MoP/MoNiP_2_, MoP/MoNiP_2_@Ti_3_C_2–_30%, MoP/MoNiP_2_@Ti_3_C_2–_60%, MoP/MoNiP_2_@Ti_3_C_2–_70%, MoP/MoNiP_2_@Ti_3_C_2–_80%, and MoP/MoNiP_2_@Ti_3_C_2–_90%) by using simple protocols. Authors reported that MoP/MoNiP_2_@Ti_3_C_2–_80% has the highest catalytic activity and enhanced PCE of 10.01% with an interesting Voc of 0.72 V and Jsc of 23 mA/cm^2^ was achieved. This PCE is higher than that of Pt (PCE = 8.22%)-based devices. Thus, it is clear that MXene-based materials may be a good choice for the fabrication of Pt-free DSSCs. In other work, Li et al. [112] also proposed a new CE material for DSSCs application. The authors prepared Fe_2_P_2_O_7_, Ni_2_P, and Fe_2_P_2_O_7_/Ni_2_P@Mxene samples as shown in Figure 6. Furthermore, authors fabricated DSSCs using Fe_2_P_2_O_7_/Ni_2_P@Mxene as CE material, and its performance was studied using J–V analysis. The Fe_2_P_2_O_7_/Ni_2_P@Mxene-80%-based DSSCs showed PCE of 9.29 % with Jsc = 17.1 mA/cm^2^, Voc = 0.76 V, and FF = 0.71. The presence of synergistic interactions in the Fe_2_P_2_O_7_/Ni_2_P@Mxene-80% resulted in enhanced photovoltaic performance compared to the other fabricated devices.

**Figure 7:**
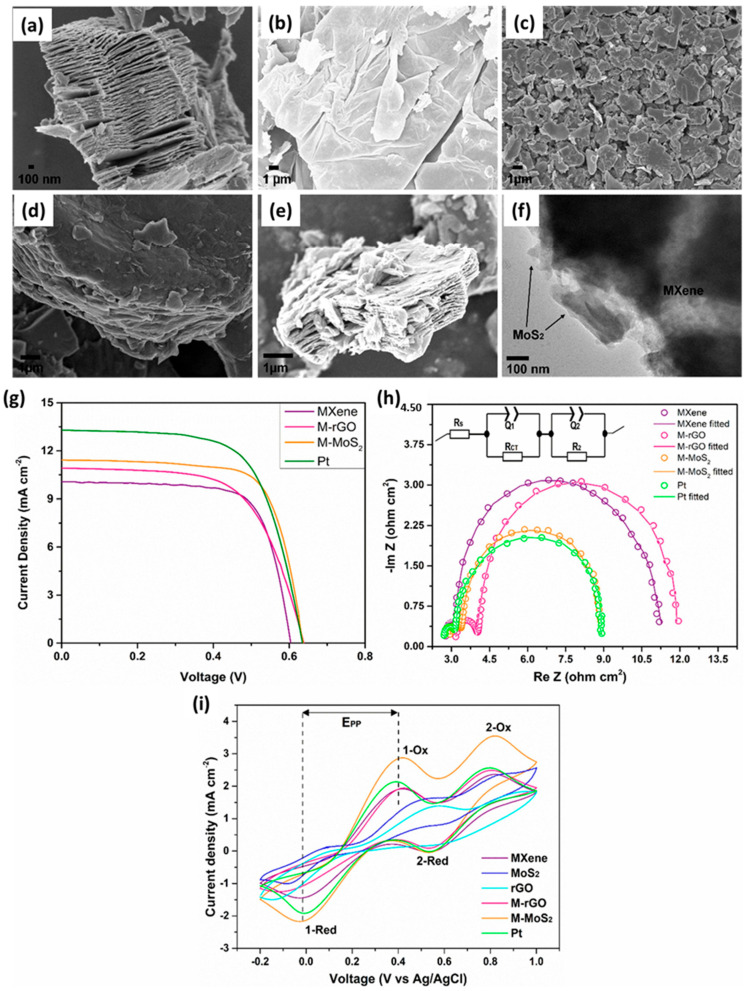
SEM images of (**a**) MXene, (**b**) rGO, (**c**) MoS_2_, (**d**) M-rGO, (**e**) M-MoS_2_, and (**f**) TEM image of M-MoS_2_. (**g**) J–V, (**h**) EIS, and (**i**) CV curves of the different CE materials. Reprinted with permission [115].

Al-Zoubi et al. [113] fabricated MXene/zinc cobaltite (Ti_3_C_2_T_x_/ZnCo_2_O_4_ (ZCO)) through solid state reaction followed by minimally intensive layer delamination (MILD) method. On the other hand, ZnCo_2_O_4_ was also synthesized by using the simple sol–gel synthesis method. The synthesized materials were characterized by various sophisticated techniques such as SEM, XRD, TEM, and EDS. The weight percentage (0%, 20%, 30%, and 40%) of the ZnCo_2_O_4_ was also optimized to enhance the catalytic properties of the desired composite material. The 30% ZnCo_2_O_4_ on Ti_3_C_2_T_x_ MXene surface demonstrated improved catalytic activity, and the fabricated device showed the PCE of 8.61%, which is higher than pristine ZnCo_2_O_4_ (5.59%)- and Pt (8.12%)-based devices. The improved performance of the proposed DSSCs was attributed to a substantial rise in current density, mainly due to the increased electro-catalytic activity of the ZM30 (30% ZnCo_2_O_4_ on Ti_3_C_2_T_x_) CE and enhanced conductivity. Hu et al. [114] reported the preparation of Ti_3_C_2_T_x_-decorated carbon nanotubes (CNTs) composite electrode materials (CNTs/Ti_3_C_2_T_x_) and tested their performance as CE in DSSC applications. The series of electrochemical tests revealed that CNTs/Ti_3_C_2_T_x_ have excellent electro-catalytic activity towards iodine-based redox electrolytes and exhibited a low charge transfer resistance (Rct) value that was close to the Pt-based CE. The CNTs/Ti_3_C_2_T_x_ (1.0 wt %) CE-based DSSCs showed PCE of 5.83%, whereas CNTs CE-based device showed PCE of 3.70%. The Pt CE-based device demonstrated PCE of 6.61%. It is clear that CNTs/Ti_3_C_2_T_x_ (1.0 wt%)-based devices have higher performance in terms of PCE compared to the CNTs or Pt based devices. The highest performance of the CNTs/Ti_3_C_2_T_x_ (1.0 wt %) based device may be attributed to the presence of synergism, conductivity, and the unique 2D structure of MXene. In addition, the photo-stability test under continuous light exposure demonstrated good stability to the electrolytes. The authors proposed that CNTs/Ti_3_C_2_T_x_ (1.0 wt%) may be promising CE material towards the development of Pt-free DSSCs. Gasso et al. [115] proposed various Pt-free CE materials for the fabrication of DSSCs. In this connection, authors prepared rGO, MoS_2_, MXene-MoS_2_ (M-MoS_2_), and MXene-rGO (M-rGO). Figure 7a shows that MXene has layered structure, whereas rGO possesses sheet-like surface morphology (Figure 7b). The synthesized MoS_2_ has a flake-like structure as shown in Figure 7c. 

The M-rGO demonstrated that rGO sheets are attached with MXene (Figure 7d). The MoS_2_ flakes were also present on the surface of MXene, as depicted in Figure 7e. The presence of MXene and MoS_2_ in the M-MoS_2_ sample was also authenticated by TEM analysis (Figure 7f). The prepared materials were further explored to fabricate the Pt-free CEs, and their performance was investigated by utilizing J–V, electrochemical impedance spectroscopy (EIS), and CV techniques. Figure 7g shows the J–V curves of the MXene, Pt, M-MoS_2_, and M-rGO CE-based DSSCs. The obtained results exhibit that a Pt-based device has a PCE of 5.5%, whereas a M-MoS_2_-based device demonstrated a PCE of 5.21%. In contrast, M-rGO-based devices showed PCEs of 4.36%, while MXene-based devices showed PCEs of 4.39%. It can be noted that M-MoS_2_-based devices have comparable performance with Pt-based devices. The EIS results also revealed that M-MoS_2_ has low Rct (Figure 7h) and higher electro-catalytic activity (Figure 7i) compared to the other CEs. This improved performance in the M-MoS_2_-based device may be attributed to the presence of synergistic interactions and 2D-2D hetero-structure.

Conducting polymers such as poly(3,4-ethylenedioxythiophene) (PEDOT) or poly(3,4-ethylenedioxythiophene) polystyrene sulfonate (PEDOT:PSS) possess excellent conductivity and catalytic properties, which makes them a decent candidate for the fabrication of conducting polymer-based CE for DSSCs. Nagalingam et al. [116] combined PEDOT polymer with MXene to develop the novel CE for the construction of DSSCs. The authors prepared PEDOT@Ti_3_C_2_T_x_ composite material using the selective chemical etching method followed by the electro-polymerization method. XRD results confirmed the formation of the PEDOT@Ti_3_C_2_T_x_ composite. The authors investigated the electro-catalytic behavior of the PEDOT@Ti_3_C_2_T_x_ composite in an iodide/triiodide electrolyte system and observed that the PEDOT@Ti_3_C_2_T_x_ composite has strong catalytic activity towards the regeneration of redox reactions. The PEDOT@Ti_3_C_2_T_x_ composite may be a promising CE material in DSSCs. Thus, authors fabricated PEDOT@Ti_3_C_2_T_x_ composite CE-based DSSCs. The fabricated DSSCs device demonstrated decent performance in terms of Voc, Jsc, and PCE. The interesting PCE of 7.12% with Jsc of 15.4 mA/cm^2^ and Voc of 0.69 V was achieved for PEDOT@Ti_3_C_2_T_x_ composite CE-based DSSCs. This PCE was higher compared to the DSSCs fabricated using MXene or PEDOT CEs. Nagalingam et al. [116] used a novel configuration of CE material towards the construction of Pt-free DSSCs. In this report, authors prepared MXene-Polythiophene (Ti_3_C_2_T_x_-P_Th_) composite by using the interfacial polymerization method. The electro-catalytic activity of the synthesized Ti_3_C_2_T_x_-P_Th_ composite was checked using CV and EIS techniques, which demonstrated the presence of excellent catalytic behavior and a low Rct value. This catalytic behavior may arise due to the presence of synergistic interactions in the prepared Ti_3_C_2_T_x_-P_Th_ composite. The authors developed DSSCs using Ti_3_C_2_T_x_-P_Th_ composite as CE, and their photovoltaic performance was evaluated by employing J–V analysis. The fabricated device exhibited an interesting PCE of 5.82%. The authors also fabricated DSSCs using Ti_3_C_2_T_x_ and P_Th_ as CE materials, and their PCE was found to be 4.61% and 4.19%, respectively. These reports suggested that PCE of the polymer-based DSSCs can be improved by combing them with MXenes. Table 2 shows the photovoltaic performance of the MXene-based DSSCs.

**Table 2:** Comparison of the photovoltaic performance of the MXenes and their composite-based DSSCs.

Material	Etching Agent or Method	Layered Structure	Jsc (mA/cm^2^)	Voc (V)	F.F.	PCE (%)	References
Ti_3_C_2_	HF	Multi-layered	15.28	0.77	0.69	8.12	[105]
Ti_3_C_2_	HF	Multi-layered	-	-	-	7.78	[106]
Ti_3_C_2_	Leaching method	Multi-layered	17.10	0.75	0.73	8.68	[107]
Ti_3_C_2_	HF	Multi-layered	13.75	0.74	0.71	7.15	[108]
Ti_3_C_2_	HF/HCl	Multi-layered	13.31	0.71	0.64	6.06	[108]
Ti_3_C_2_	LiF/HCl	Multi-layered	13.38	0.73	0.65	6.38	[108]
Ti_3_C_2_	NH_4_HF_2_	Multi-layered	11.81	0.72	0.71	6.05	[108]
TiN@Ni-MXene	Impregnation method	Multi-layered	15.91	0.76	0.66	8.08	[109]
CoMoP_2_@Mxene-3	HF	Multi-layered	15.17	0.73	0.63	7.08	[110]
CoMoP_2_@Mxene@CNTs-3	HF	Multi-layered	25.43	0.70	0.59	10.64	[110]
MoP/MoNiP_2_@Ti_3_C_2_–80%	HF	Multi-layered	23.0	0.72	0.62	10.01	[111]
Fe2P_2_O_7_/Ni_2_P@Mxene-80 %	HF	Multi-layered	17.1	0.76	0.71	9.29	[112]
ZM30	LiF/HCl	Multi-layered	17.53	0.72	0.68	8.61	[113]
C/T-1.0 wt%	LiF/HCl	Multi-layered	13.57	0.71	0.60	5.83	[114]
M−MoS_2_	HF	Multi-layered	11.35	0.64	0.71	5.21	[115]
PEDOT@ Ti_3_C_2_T_x_	HF	Multi-layered	15.40	0.69	0.67	7.12	[116]
Ti_3_C_2_T_x_-P_th_	HF	Multi-layered	15.54	0.67	0.56	5.83	[117]

## 5. Conclusions

It is well known that 2D-layered MXenes exist in single and multi-layered structures. Single-layered MXenes have interesting properties such as high specific surface area, conductivity, and electrochemical reactivity for redox reactions. However, single-layered MXenes have low stability compared to multi-layered MXenes. In the past years, MXenes have been widely used as electrode material for the development of electrochemical sensors and DSSC applications. It is clear that MXene plays a significant role as electrode material for the construction of electrochemical sensors and DSSCs. In both applications, MXenes acts as an electro-catalyst and improves charge transport. It has been observed that multi-layered MXenes or its composite materials have been widely used as electrode materials for electrochemical sensing as well as DSSC applications. The multi-layered MXene-based electrode materials demonstrated excellent stability and sensitivity for the determination of phenolic compounds. However, the sensing activity of the MXene-based materials can be further improved by developing more sensitive methods compared to the CV or LSV. The CV and LSV are widely used simple methods for the sensing of various analytes, but these techniques are less sensitive in some cases. Additionally, MXene-based electrode materials can be combined with enzymes to further enhance the activity of the electrode modifiers. It has been found from the published reports that DPV and SWV techniques for the determination of phenolic compounds using MXene or MXene-based composites as electrode modifiers. It was found that the DPV method is more sensitive compared to the LSV, CV, or SWV. Many studies reported excellent LOD, wide dynamic linear range, and robust stability using the DPV method. Thus, it is expected that DPV may be the most efficient analytical technique for the determination of phenolic compounds. However, some drawbacks may arise, such as some derivatives having similar oxidation peaks with a small change in the potential. Thus, selectivity in such cases may be compromised. For this reason, the amperometric approach may be a promising method for the selective sensing of phenolic compounds and their derivatives. The MXene itself has excellent conductivity and is a promising electrode material. However, its combination with MOF and layered double hydroxide may boost their performance for the fabrication of highly efficient electrochemical sensors. On the other side, DSSCs are the most promising photovoltaic devices due to their excellent stability in ambient conditions compared to other devices such as perovskite solar cells. However, the PCE of the DSSCs is still lower, which needs significant improvements. In addition, Pt is the most expensive metal, which needs to be replaced with low-cost and environmentally friendly metals. The PCE of the Pt-free DSSC devices should be improved. In the past decades, many counter-electrode materials based on metal oxides, polymers, MOFs, and various 2D-2D composite materials have been reported. Unfortunately, PCE of the reported DSSC devices is still far, and most of the devices exhibited lower PCE compared to the Pt-based devices. MXene-based composite materials demonstrated good performance in terms of PCE, which is comparable with Pt-based devices. The MXene/polymers exhibited lower performance, but MXene/MoNiP_2_ type materials demonstrated higher PCE values. We believe that PCE of the MXene composite CE-based DSSCs can be further improved by combining MXenes with MOFs or COFs. Moreover, some efficient photo-anodes need to be developed for further enhancements in the photovoltaic performance of the Pt-free DSSCs.

### 5.1. Environmental Implications

The environmental implications of using MXenes or their composites should be carefully considered in a real-world scenario to balance the benefits with sustainable and potential aspects. The MXenes are widely used for a variety of applications such as energy storage, catalysis, water purifications, batteries, fuel cells, and photovoltaics. Thus, it is expected that MXenes or their composite-based materials can contribute positively towards the development of clean energy technologies and may address the environmental challenges related to water contamination by promoting sustainable clean water solutions. Despite a positive response of the MXene-based materials, the toxicity of the etching agents (HF) and the presence of functional groups (-F) may be the concern for environment-related issues. The HF may result in severe injuries or environmental contamination. Thus, it is required to find out novel HF-free etching agents or less toxic solvents to prepare the high-quality MXenes. The life cycle analysis, recycling, and safe disposal of the harmful byproducts/etching agents should be carefully carried out. In the future, it may be expected that toxicity may be reduced by tailoring the surface functional groups of the MXenes without comprising their properties. The biocompatible MXenes may be used in drug delivery systems and biomedical applications.

### 5.2. Challenges, Limitations, and Future Perspectives

The MXenes have been synthesized by various methods in the laboratories. However, it still needs significant efforts to prepare the MXenes using scalable and environmentally friendly approaches for large-scale production. The current methods have various limitations, such as the use of HF etchant, which is toxic and corrosive and poses hazardous effects on the environment and human safety. In the present scenario, most of the existing synthesis approaches rely on batch processing, which poses challenges for scalability. The continuous production techniques, crucial for industrial applications, are still underdeveloped in the context of MXene synthesis. Additionally, etching and delamination processes consume significant energy and a large quantity of water, which may raise production costs and contribute to a larger environmental impact. It is a challenging task to maintain the quality of the MXenes, such as layer thickness, surface functionalization, etc., for scaling-up production. In addition to the scalability, the environmental implications of MXene synthesis are an important factor to consider. Optimizing synthesis methods for both scalability and environmental sustainability is very essential for the large-scale industrial production of MXenes. Overcoming challenges such as the use of hazardous chemicals, scaling up production, and ensuring high-quality output demands innovative approaches and collaboration across disciplines. It can be considered that by developing more eco-friendly synthesis methods, improving production efficiency, and ensuring consistent quality, MXenes can fully realize their potential in a range of high-impact applications while minimizing environmental and safety concerns. It is also necessary to explore specialized techniques for accurately controlling the surface terminations of MXenes to achieve desired performance for various applications. Additionally, further research into post-treatment methods, such as alkali and heat treatments, is needed to modify MXene surfaces and enhance their performance and stability. The integration of theoretical calculations with experimental research should be improved to gain a deeper understanding of how various surface terminations affect MXene performance, helping to close the gap between theory and practical experimentation. Eco-friendly and efficient synthesis methods for MXenes should be investigated to minimize environmental impact and improve production efficiency. In addition, degradation of MXenes, in particular for Ti₃C₂Tₓ, may involve the oxidation process, which leads to the formation of TiO₂ at the edges of the flakes. Zhang et al. [18] reported that degradation of MXenes started at the edges of the MXene and called it the scissor effect, which shreds the MXene nanosheets into smaller particles. This degradation process may be significantly accelerated in the presence of dissolved oxygen or water. Thus, it is clear that MXene may be converted to metal oxide if it is thermally heated in the presence of oxygen. For DSSCs applications, MXene may be coated on working electrodes in presence of nitrogen or inert conditions. The oxidation of MXene may be further impacted by the size of the nanosheets, and smaller nanosheets may degrade fast compared to the larger nanosheets due to their higher edge-to-area ratio. Zhang et al. [18] reported that degradation of MXenes may be reduced by storing MXenes in argon gas-filled vials at low temperature. Another approach is to filter the MXene colloidal solution to form films, which can be re-dispersed as required and may help to maintain the flakes’ quality over time.

## Data Availability

No data was generated and authors are unable to share the data.
